# Reverse Transcription in the *Saccharomyces cerevisiae* Long-Terminal Repeat Retrotransposon Ty3

**DOI:** 10.3390/v9030044

**Published:** 2017-03-15

**Authors:** Jason W. Rausch, Jennifer T. Miller, Stuart F. J. Le Grice

**Affiliations:** Reverse Transcriptase Biochemistry Section, Basic Research Laboratory, Frederick National Laboratory for Cancer Research, Frederick, MD 21702, USA; rauschj@mail.nih.gov (J.W.R.); millerj@mail.nih.gov (J.T.M.)

**Keywords:** retrotransposon, Ty3, reverse transcriptase, reverse transcription, ribonuclease H (RNase H), DNA polymerase, retroelement

## Abstract

Converting the single-stranded retroviral RNA into integration-competent double-stranded DNA is achieved through a multi-step process mediated by the virus-coded reverse transcriptase (RT). With the exception that it is restricted to an intracellular life cycle, replication of the *Saccharomyces cerevisiae* long terminal repeat (LTR)-retrotransposon Ty3 genome is guided by equivalent events that, while generally similar, show many unique and subtle differences relative to the retroviral counterparts. Until only recently, our knowledge of RT structure and function was guided by a vast body of literature on the human immunodeficiency virus (HIV) enzyme. Although the recently-solved structure of Ty3 RT in the presence of an RNA/DNA hybrid adds little in terms of novelty to the mechanistic basis underlying DNA polymerase and ribonuclease H activity, it highlights quite remarkable topological differences between retroviral and LTR-retrotransposon RTs. The theme of overall similarity but distinct differences extends to the priming mechanisms used by Ty3 RT to initiate (−) and (+) strand DNA synthesis. The unique structural organization of the retrotransposon enzyme and interaction with its nucleic acid substrates, with emphasis on polypurine tract (PPT)-primed initiation of (+) strand synthesis, is the subject of this review.

## 1. Introduction

Central to the propagation of retroviruses and long terminal repeat (LTR)-retrotransposons is the conversion of their single-stranded RNA genome into integration-competent double-stranded DNA, a multi-step process mediated by the element-encoded reverse transcriptase (RT) [1]. Crucial steps in this process involve the use of RNA primers to initiate synthesis of the (−) and (+) strand DNAs (a host-coded transfer RNA (tRNA) and the element-encoded polypurine tract (PPT), respectively). Our understanding of these events has come almost exclusively from retroviruses where, over some 50 years, the field has witnessed a progression from the discovery of an enzyme capable of synthesizing *DNA* on an RNA template [2,3] to high resolution X-ray structures for human immunodeficiency virus type 1 (HIV-1) RT that have proven instrumental to the success of combination antiviral therapy to stem HIV infection and the progression of acquired immunodeficiency syndrome (AIDS) [1].

Based on literature that has been amassed on RT from human, avian and murine retroviruses, it might be considered reasonable to assume that counterpart enzymes of transposable elements (e.g., *Drosophila* (*copia*) and *Saccharomyces cerevisiae* (Ty1 and Ty3))*,* as well as their cognate nucleic acid substrates, are merely minor variations of a common theme. However, the observation that (a) Ty1 and Ty3 RTs use a bipartite primer binding site (PBS); (b) the *Schizosaccharomyces pombe* element Tf1 uses a tRNA-independent mechanism; and (c) a “half-tRNA” is employed by *Drosophila melanogaster copia* to initiate (−) strand DNA synthesis [4] suggests their respective polymerases might also not share the topological features of HIV-1 RT. This issue is highlighted by structural data for several monomeric retroviral and retrotransposon RTs such as the gammaretroviruses xenotropic murine leukemia virus-related virus (XMRV) and Moloney murine leukemia viruses, mouse mammary tumor virus, simian foamy virus, bovine leukemia virus, and the Tf1 element [5,6,7,8,9,10]. As the third RNA-dependent DNA polymerase to be crystallized in the presence of an RNA/DNA hybrid, the goal of data presented in this review is to illustrate the unique topological complexity of Ty3 RT and point out to the reader that our understanding of reverse transcription should be the consequence of comparative studies and not simply those from a single enzyme.

## 2. Reverse Transcription Overview

**Figure 1:**
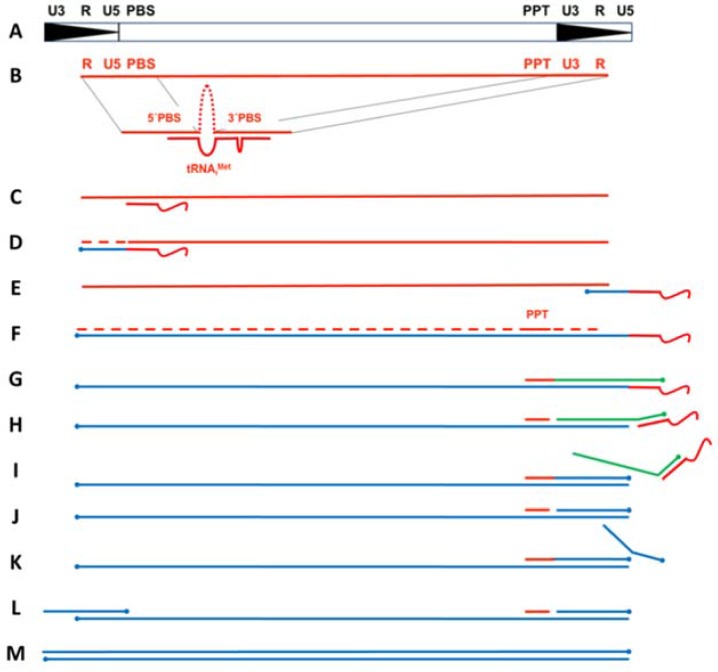
Ty3 Reverse Transcription Cycle. (**A**) Structure of the double stranded preintegrative Ty3 DNA (black). U3, unique 3′ sequence; R, repeat sequence; U5, unique 5′ sequence; PBS, primer binding site; PPT, polypurine tract; (**B**) Genomic RNA is depicted in red. The bipartite nature of the PBS comprises sequences from both the 5′ PBS and the 3′ U3 regions; (**C**) Simplified initiation complex excluding the transfer RNA (tRNA) 5′ terminal nucleotides; (**D**) (−) strand strong stop synthesis, with concomitant degradation of genomic RNA by RNase H. Newly synthesized (−) strand DNA is shown in blue; (**E**) (−) strand transfer; (**F**) (−) strand synthesis and concomitant degradation of genomic RNA by RNase H; (**G**) (+) strand synthesis initiates from the PPT and extends into tRNA. Nascent (+) strand DNA is shown in green; (**H**) PPT is re-cleaved from (+) strand DNA and tRNA is cleaved from (−) strand DNA by RNase H; (**I**) Second (+) strand DNA, indicated in blue, displaces first; (**J**) PPT is again cleaved; (**K**) Third (+) strand synthesis initiates, and displaces second (+) strand; (**L**) Second (+) strand transfers to 3′-end of (−) DNA and PPT is cleaved; (**M**) Synthesis of both (+) and (−) strands is completed.

Ty3 RT performs a series of orchestrated events to convert the diploid plus (+) stranded retrotransposon RNA into double-stranded DNA (dsDNA) that is subsequently integrated into the host cell genome (Figure 1). Minus (−) strand DNA synthesis initiates from the 3′-end of a host-derived tRNA hybridized to a bipartite primer binding site (PBS) and continues until the 5′-end of the genome is reached (Figure 1B–D). RT-associated RNase H activity then hydrolyzes the 5′-terminal repeat (R) and U5 segments of the RNA template, allowing transfer of the nascent (−) strong stop DNA (ssDNA) to the 3′-terminal R segment (Figure 1D,E). After the template switch, minus (−) strand DNA synthesis proceeds with concomitant RNase H-mediated degradation of viral RNA, leaving a small RNase H-resistant purine-rich RNA fragment (polypurine tract, or PPT) hybridized to the nascent DNA (Figure 1F,G). In contrast to retroviruses and Ty1, no central PPT has been identified for Ty3. The Ty3 PPT fragment primes (+) strand DNA synthesis in a manner that diverges somewhat from the equivalent event in retroviruses. Through a mechanism that will be discussed in more detail below, the (+) strong stop DNA generated from a second PPT priming event is transferred to the 3′-end of the nascent (−) DNA by virtue of the terminal repeat (R) sequences. Once both the (+) and (−) strands are filled out, the final dsDNA contains a repeated U3-R-U5 sequence flanking the coding regions of the retrotransposon genome (Figure 1G–M).

### 2.1. Minus (−) Strand Initiation and tRNA-Retrotransposon RNA Interactions

Minus (−) strand DNA synthesis in Ty3 is primed by host tRNA_i_^Met^, the same species utilized by the distantly-related Ty1 and Ty5 retrotransposons [11]. Interestingly, while Ty3 and Ty1 prime from the native 3′-end of the tRNA, Ty5 RT initiates from a 3′-end produced by host cell RNase P-mediated internal cleavage within the anticodon loop [12,13,14]. Also, both Ty3 and Ty1 utilize a bipartite PBS, although the details of how the PBS is divided and where the segments reside in their respective RNA genomes differs between the two elements. Like those of retroviruses, the PBS of Ty1 is contained entirely within U5, with the two segments separated by a relatively small internal loop. In contrast, the 5′ and 3′ segments of the bipartite Ty3 PBS are separated by ~4800 nt and reside in the 5′ (PBS) and 3′ (U3) untranslated regions (UTRs), respectively. To form a DNA synthesis-competent initiation complex, the acceptor stem and TΨC arm of tRNA_i_^Met^ hybridize to the 5′ and 3′ components of the PBS while the D arm interacts with viral RNA in U3 [15,16]. Such intricate interactions to establish (−) strand initiation complexes are a common requirement for many retroelements, including retroviruses. For instance, mutational analyses of HIV-1, feline immunodeficiency virus (FIV) and Rous sarcoma virus (RSV) complexes indicate that base pairing between tRNA and viral RNA sequences outside of the PBS support an efficient transition from the initiation to elongation phase of DNA synthesis [17,18,19,20,21,22].

Ty3 nucleocapsid protein (NC) is produced by proteolytic cleavage of the *GAG3* (CA-SP-NC) precursor [23]. Ty3 NC has a single zinc finger, the highly-basic *N*-terminal domain of which contributes to nucleic acid binding efficiency [13,24], facilitating annealing of tRNA_i_^Met^ to the PBS, formation of ribonucleoprotein complexes, and genomic RNA dimerization. Deletion analysis has determined that these NC functions are more dependent on the basic region than the zinc finger [15]. Together with tRNA_i_^Met^-PBS hybridization, Ty3 NC enables initiation complex dimerization by promoting interstrand base pairing between 12 nt G:C rich palindromic sequences at the tRNA 5′-ends [13]. One study also suggests that a global complex in which viral RNA 5′ and 3′ termini are brought into proximity may be stabilized by a transient covalent linkage between the two ends, as knockdown mutations in the lariat debranching enzyme Dbr1 have significantly decreased levels of Ty3 cDNA accumulation [25].

### 2.2. Plus (+) Strand Initiation, (+) sssDNA Synthesis, and (+) Strand Transfer

Plus-strand synthesis in Ty3 initiates from a PPT RNA fragment located just upstream of U3. However, in Ty3 and Ty1, this PPT appears to prime DNA synthesis more than once. This revelation came from experiments in which a mutant tRNA was used to prime minus-strand initiation, yet this change was not reflected in the PBS region of Ty1 or Ty3 DNA following retrotransposition [26].

In these experiments, researchers utilized a mutant yeast strain devoid of any endogenous tRNA_i_^Met^ genes but expressing a similar mutant tRNA containing a nucleotide substitution in the anti-PBS sequence [27]. After performing a Ty3-specific integration assay, progeny retrotransposon DNA was sequenced and did not contain the mutation, indicating the genomic PBS sequence could not be derived from reverse transcription of the (−) DNA-priming tRNA, as is the case in retroviruses. The authors proposed the alternative PPT recycling mechanism shown schematically in Figure 1. In this process, (+)-DNA synthesis initiates from the PPT and terminates after reverse transcribing 12 nt of the (−)-strand priming tRNA (Figure 1G). RT then separates the tRNA from the (−) DNA template by cleaving at or near the tRNA-DNA junction. RT also cleaves at the junction between the PPT and nascent (+) DNA, after which synthesis of a second (+) strand initiates from the regenerated 3′PPT primer, displaces the first (+) strand strong stop DNA (sssDNA), and terminates at the end of U5 (since the tRNA has been removed from the (−) DNA template) (Figure 1H,I). Finally, a third cleavage of the PPT allows re-initiation of a third (+) DNA synthesis product, resulting in displacement of the second (+) sssDNA, and making it available for hybridization to the complementary R and U5 sequences at the (−) DNA 3′ terminus (Figure 1J–L). Re-initiation of (−) DNA synthesis from the transferred strand completes the (+) strand transfer process (Figure 1L,M).

As the alternative (+) DNA synthesis mechanism would suggest, dead end (+) sssDNA products have been found to accumulate to high levels in Ty3 virus like particles [28]. This observation, together with finding that the PBS sequence is not preserved by reverse transcription of the tRNA 3′ terminus, lends support to this distinctive and intriguing model of (+) strand synthesis and strand transfer.

### 2.3. Involvement of Ty3 Integrase

Ty3 integrase (IN) is produced by proteolytic cleavage of the polyprotein precursor *GAG3-POL3* (PR-J-RT-IN). [23,29] To determine whether this enzyme might affect stages of retrotransposition outside of integration, researchers substituted alanine for charged non-catalytic residues in both the *N*- and *C*-terminal domains of Ty3 IN and studied the effects in vivo. One class of such mutations that reduced steady state levels of IN in cells also produced a correlative decrease in accumulated cDNA. Similarly, mutant virus-like particles (VLPs) contained less primer tRNA and produced less (−) sssDNA in exogenous RT assays, suggesting IN may contribute a stimulatory role at early stages of reverse transcription. *Trans*-complementation with a capsid (CA)-RT-IN, but not a CA-IN construct, rescued cDNA production, indicating that the stimulatory effects of IN on cDNA synthesis may be mediated by close association of this enzyme with RT [30]. Ty1 experiments in which native IN was provided in *trans* yielded similar results wherein *trans*-complementation of IN alone failed to rescue reverse transcription defects in an IN-deficient Ty1 model system [31]. Taken together, these studies suggest that the mechanism of activating initiation of (−) DNA synthesis by association of IN with RT may be common among retrotransposons.

## 3. Ty3 RT Structural Organization and Biochemical Characterization

The reverse transcription process has been thoroughly characterized for several retroviruses and LTR-containing retrotransposons. In contrast, high resolution structural details on their associated RTs have been limited largely to the HIV-1 enzyme as a consequence of its central role as an antiviral target [32]. In the absence or presence of its nucleic acid substrate, HIV-1 RT is organized as an asymmetric heterodimer of 66 and 51 kDa subunits (p66 and p51, respectively) derived from the same gene, but differing in that p51 lacks the ~15 kDa, *C*-terminal RNase H domain as a consequence of processing by the virus-coded protease [33]. Similar to other nucleic acid polymerases, p66 subdomains were designated “fingers”, “palm”, and “thumb”, which were tethered to the *C*-terminal RNase H domain via a “connection” subdomain. Alternative folding of the p51 subunit positioned the connection between its fingers and palm, thereby occluding its DNA polymerase active site [34]. The lack of a p51-associated RNase H domain thus indicated that both the polymerizing and hydrolytic activities of HIV-1 RT were a property of the p66 subunit.

Later studies with RT from the gammaretrovirus xenotropic murine leukemia virus-related virus (XMRV) [5] demonstrated a monomeric organization in the absence and presence of nucleic acid substrate, providing a second example of a retroviral polymerase whose dual enzymatic functions reside on the same subunit. The availability of high resolution structures for two retroviral enzymes in the presence of an RNA/DNA hybrid thus predicted that their LTR-retrotransposon counterpart would assume one of these two configurations. Initial clues that this might not be so simple came from phylogenetic studies indicating that LTR-retrotranspon RT lacks a “connection” subdomain (i.e., its RNase H and DNA polymerase domains domain were juxtaposed) [35]. Initial biochemical characterization of recombinant Ty3 RT indicated that, following gel permeation chromatography, DNA polymerase activity was associated with a polypeptide that migrated consistent with 55 kDa monomer [36]. However, when the same analysis was conducted in the presence of nucleic acid, the migration properties of the nucleoprotein complex, 125 kDa, suggested the intriguing notion of substrate-dependent dimerization [37], in this case a homodimer. However, in contrast to HIV-1 RT, the Ty3 homodimer would retain two copies of the *C*-terminal RNase domain, raising speculation that both might exhibit activity. Our high resolution structure of Ty3 RT containing an RNA/DNA hybrid derived from its PPT answered this question, while at the same time it also demonstrated a uniquely versatile enzyme with respect to subunit topology.

**Figure 2:**
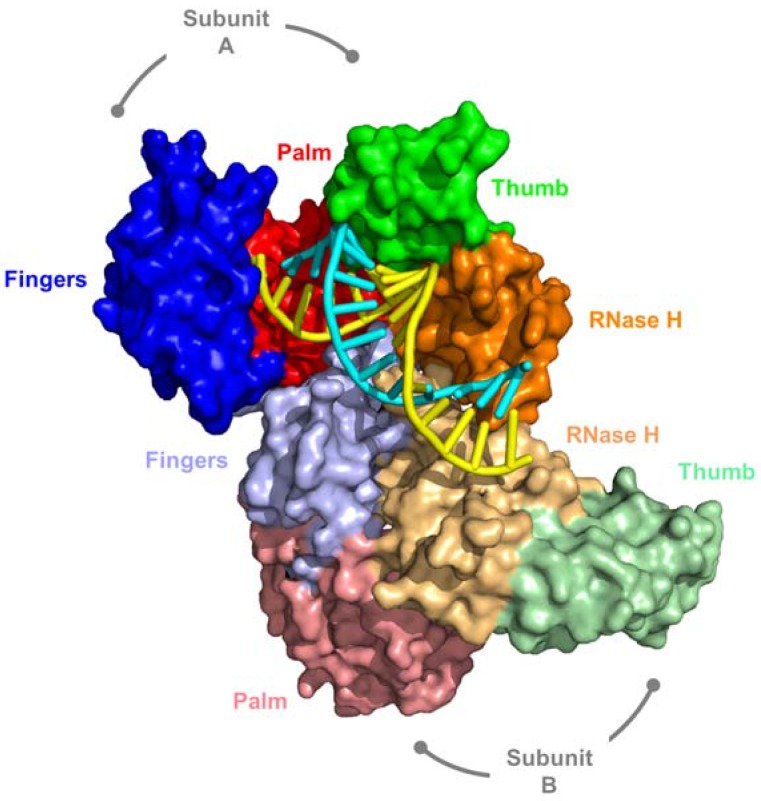
Structure of the asymmetric Ty3 RT homodimer in complex with its PPT-containing RNA/DNA hybrid. DNA and RNA strands of the cartoon representation are denoted in cyan and yellow, respectively. Subunit domains are color coded blue, red, green, and orange for fingers, palm, thumb, and RNase H, respectively, and the darker shading represents subunit A. Note the absence of a connection subdomain, a significant contrast between retroviral and LTR-retrotransposon RTs. Adapted from [37].

**Figure 3:**
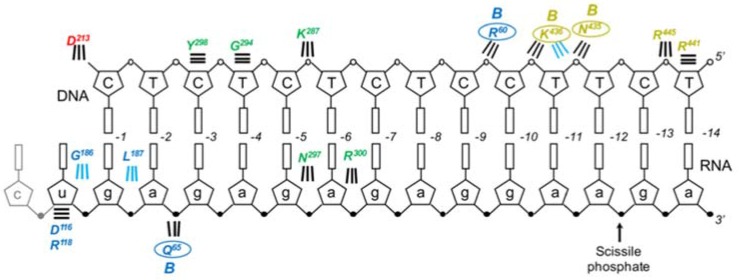
Contacts between Ty3 RT subunits A and B and the PPT-containing RNA/DNA hybrid. Color coding is consistent with subdomain designation of Figure 2, and DNA and RNA nucleotides are denoted in capital and small letters, respectively. The scissile PPT/U3 junction has been indicated, and base numbering is relative to substrate bound at the DNA polymerase active site Subunit B contacts are denoted “B” and circled. Parallel horizontal lines indicate van der Waals interactions. Diagonal and vertical lines indicate interactions mediated by the protein backbone (cyan) or side chains (black).

As depicted in Figure 2, Ty3 RT is an asymmetric homodimer comprised of subunits we designated A and B. In contrast to the previous studies of Sarafianos et al. [38], but in keeping with our own data for HIV-1 [39,40] and XMRV RT [5], the RNA/DNA hybrid assumes a more A-like configuration, displaying no steric clashes between O2′ and O4′ oxygens of adjacent riboses of the RNA strand. Although lacking a connection subdomain, the fingers, palm, thumb, and RNase H domain of Ty3 RT subunit A are topologically similar to those of HIV-1 RT p66. In addition to crystallographic data in the presence of an RNA/DNA hybrid, ascribing DNA polymerase function exclusively to subunit A was based on the observation that alternative folding positioned the subunit B RNase H domain between its fingers and palm. Thus, despite major structural differences between HIV-1 and Ty3 RT, they share the common property that alternative folding of the two subunits occludes one of the DNA polymerase active sites. A summary of amino acid contacts supported by subunits A and B is illustrated in Figure 3.

### 3.1. DNA Polymerase Active Site Residues

**Figure 4:**
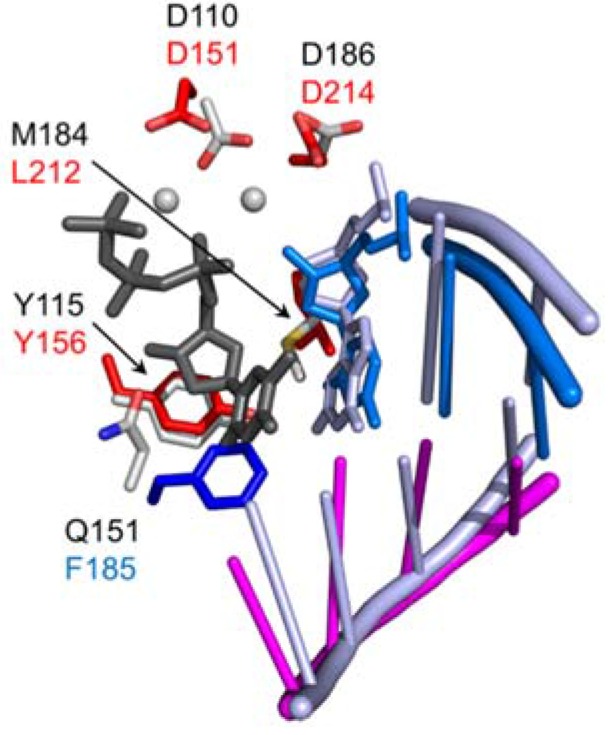
Alignment of the DNA polymerase active sites of Ty3 (PDB ID 4OL8, REF) and HIV-1 RT (PDB ID:1RTD). Carbon atoms of select Ty3 RT residues are shown in red (palm) and blue (fingers), and those of HIV-1 residues are in grey. The two catalytic metal ions and incoming dTTP are shown in grey and dark grey, respectively. Both HIV-1 DNA strands are shown as a light blue ladder, and the RNA template and DNA primer bound by Ty3 RT are shown in magenta and marine, respectively. The 3′-terminal nucleotides in both DNA primer strands are shown in stick form, and the stick radius of the incoming dTTP has been slightly expanded for contrast. Adapted from [37].

As originally identified by homology with HIV-1 RT, D151, D213, and D214 are housed in the palm subdomain and comprise the catalytic triad of the -d-(aa)_n_-Y-l-d-d- DNA polymerase active site of Ty3 RT [41] (Figure 4). These residues were mutated to either asparagine or glutamate and the effects on enzyme function were determined in vitro in the context of purified enzyme as well as transposition activity in *S. cerevisiae*. D151N and D213N substitutions eliminated both RNA-dependent and DNA-dependent DNA polymerase activities, whereas activity was retained in D214N and D214E mutants (although enzyme processivity was substantially reduced). D151E mutants were likewise devoid of polymerase activity, although D213E was partially tolerated. Reduced pyrophosphorolysis activity was found to parallel DNA polymerase activity deficits, and none of these mutants were substantially rescued by substituting MnCl_2_ for MgCl_2_ in enzyme assays. Quantitative kinetic analysis indicated that the principle effects of these mutations were on turnover and processivity rather than substrate binding.

In vitro, D151E RT was only 2% active relative to the wild type enzyme. All other mutants were at least 25% active, indicating that they were not structurally compromised and still capable of substrate binding. Both wild type and mutant enzymes retained the precision of RNase H activity, indicating that active site residues do not affect positioning of the enzyme on the substrate. In vivo, all mutations proved lethal for transposition. Taken together, these results suggested that D151 and D213 were required for coordination of the catalytically essential divalent Mg^++^, while D214 may stabilize the polymerase activation complex or otherwise facilitate catalytic chemistry. The Ty3 RT-RNA/DNA co-crystal structure also shows that, in addition to its role in metal ion chelation, the D213 side chain also contacts the 3′-terminal nucleotide of the DNA primer [37].

### 3.2. Thumb Subdomain Residues Contacting Nucleic Acid

In retroviral RTs and other DNA polymerases, the thumb subdomain is flexible and, in the context of an active polymerase domain, functions both in substrate binding and translocation during DNA synthesis [42]. Numerous residues in the Ty3 subunit A thumb contact either the primer or template strand in the RT-RNA/DNA co-crystal [37]. Specifically, DNA primer nucleotides at positions −3 to −5 form backbone contacts with thumb residues Y298, G294, and K287, respectively, while N297 and R300 contact the 2′OH moiety of the RNA strand at positions −5 and −6. Equivalent residues in the B subunit do not contact nucleic acid, as the thumb subdomain is displaced from the palm and rotated relative to the RNase H domain. Before the high resolution crystal structure became available, thumb residues proposed to interact with the nucleic acid substrate were identified by homology to the equivalent domain in HIV-1 RT [43]. On this basis, residues Q290, F292, G294, N297, and Y298 were subjected to mutational and biochemical analysis to characterize their roles in enzyme function.

A novel assay developed for this study utilized duplex DNA substrates containing serial locked nucleic acid (LNA) substitutions in either the primer or template strand [43,44]. Because LNA can only assume an RNA-like C3′-endo sugar pucker and contains a methylene bridge between ribose 2’-O and 4’-C atoms, its introduction into DNA creates a localized steric barrier to polymerase binding and/or translocation. Moreover, because only the ribose groups of LNAs are chemically modified, measuring the efficiency of single nucleotide incorporation in these substituted substrates can be exploited to determine contact sites between the enzyme and sugar-phosphate backbone irrespective of nucleoside base identity.

In this assay, LNA substitutions at either position −3 or −4 in the DNA primer strand or position −6 or −7 in the DNA template strand impaired single nucleotide incorporation, indicating the importance of enzyme-nucleic acid contacts at these sites for proper substrate binding. This finding was corroborated by parallel assays in which a basic nucleoside analogs were serially substituted into nucleic acid substrates, and is in remarkable agreement with the high resolution Ty3 RT-RNA/DNA co-crystal structure published nine years later [37]. Analysis of Ty3 RT thumb mutants using this assay indicated that subunit A residues G294, N297, and Y298 contact the DNA substrate at or near the sites indicated in the co-crystal structure. Perhaps the most remarkable finding was the compensatory interaction between the Y298A mutant and the DNA substrate with an LNA substitution at primer nucleotide −3. Primer extension activity of this mutant was substantially greater than that of wild type Ty3 RT, indicating a reciprocally favorable binding interaction between the smaller Ala side chain and the bulky modified nucleoside.

The important contribution of thumb contacts to Ty3 RT function was further established by more conventional biochemical assays [43]. Higher rates of dissociation from duplex DNA substrates were measured in steady-state kinetic assays, while mutants containing G294, N297, or Y298 substitutions exhibited reduced RNase H activity.

### 3.3. A Single Subunit of the Ty3 RT Asymmetric Homodimer Contributes to RNase H Activity

Although contacts with the DNA strand of the RNA/DNA hybrid could be identified for both RNase H domains in the crystal structure, neither RNase H active site was in the vicinity of the RNA scissile bond. Since simple site-directed mutagenesis would duplicate any modification in both subunits, the origin of RNase H activity was determined using a novel phenotypic mixing strategy in which the nucleoprotein complex was reconstituted with selectively-deficient Ty3 RT monomers.

Residue D426 constitutes one of the catalytically critical residues of the RNase H domain, and its replacement with asparagine (N426) was shown to eliminate RNase H activity [45]. The capacity of this variant to dimerize, however, appears to be unaffected, as the D426N enzyme was fully functional as a DNA polymerase. In contrast, R140 and R203 of Ty3 RT subunit A localize to the dimerization interface, suggesting that mutating these residues might impair dimerization, and hence enzyme function. Indeed, an R140A/R203A double mutant was defective in both DNA polymerase and RNase H activities, presumably reflecting a failure to dimerize. It is important to note that these mutations only prevent dimerization when present in the context of the A subunit; in the B subunit, residues R140 and R203 do not appear to be directly involved in dimerization or any other aspect of RT function.

**Figure 5:**
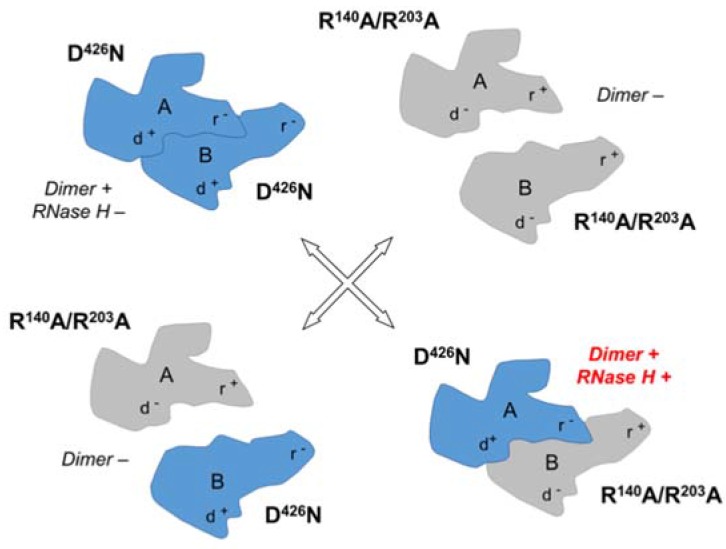
Phenotypic mixing strategy to determine the RNase H-competent Ty3 RT subunit. RNase H defective (D426N) and dimerization defective (R140A/R203A) mutant monomers are indicated in blue and grey, respectively. Notations d^+^ and d^−^ indicate a dimerization-competent and dimerization-incompetent subunit interface, while r^+^ and r^−^ denote RNase H-competent and RNase H-incompetent, respectively. Note that the d^−^ mutant only prevents dimerization when in the A subunit position. When purified mutants are mixed, RNase H activity is only recovered in a reconstituted dimer whose subunit B contributes to RNase H activity.

The possible complementation outcomes of the mixing of D426N and R140A/R203A Ty3 RT monomers are depicted in Figure 5. In brief, the only way for these variants to combine to form an active dimer with RNase H activity would be if (i) mutants D426N and R140A/R203A occupied the subunit A and B positions, respectively; and (ii) the RNase H domain of subunit B confers RNase H activity to Ty3 RT. This was indeed what we observed experimentally [37], demonstrating that DNA polymerase and RNase H activity are exclusive to the A and B subunits of Ty3 RT, respectively. An unresolved question, however, was the conformational change necessary to position the subunit B active site in the vicinity of the scissile bond of the RNA backbone. Although located closer to the scissile phosphate, the subunit B RNase H domain (and thumb subdomain) would be required to move ~40 Å, a translation molecular modeling suggests could be accommodated for without invoking steric clashes. In summary, although the active site residues of DNA polymerase domains of lentiviral, gammaretroviral, and LTR-retrotransposon RTs are well conserved, the major differences they exhibit in the topology of their RNase H domains possibly reflect an intricate evolutionary mechanism whereby cellular RNases H were sequestered by the retroviral polymerase into bifunctional enzymes.

### 3.4. RNase H Domain Structure

Retroviral, bacterial, human H1, and Ty3 RNase H enzymes/domains adopt a common “RNase H fold” characterized by a 5-stranded β-sheet flanked by 2–3 α-helices on one side and one on the other [46]. Aside from their positioning relative to nucleic acid substrate and the Ty3 fingers, palm, and thumb subdomains, the Ty3 RNase H domain differs from the retroviral and RNase H1 counterparts in the length of the first β-strand (~10 residues shorter for Ty3 RT) and arrangement of α-helices between β-strands 4 and 5. Secondary, tertiary, and quaternary structures of Ty3 RNase H domains also resemble the connection subdomains of closely related retroviral enzymes, although the latter elements lack the functional catalytic residues [35].

**Figure 6:**
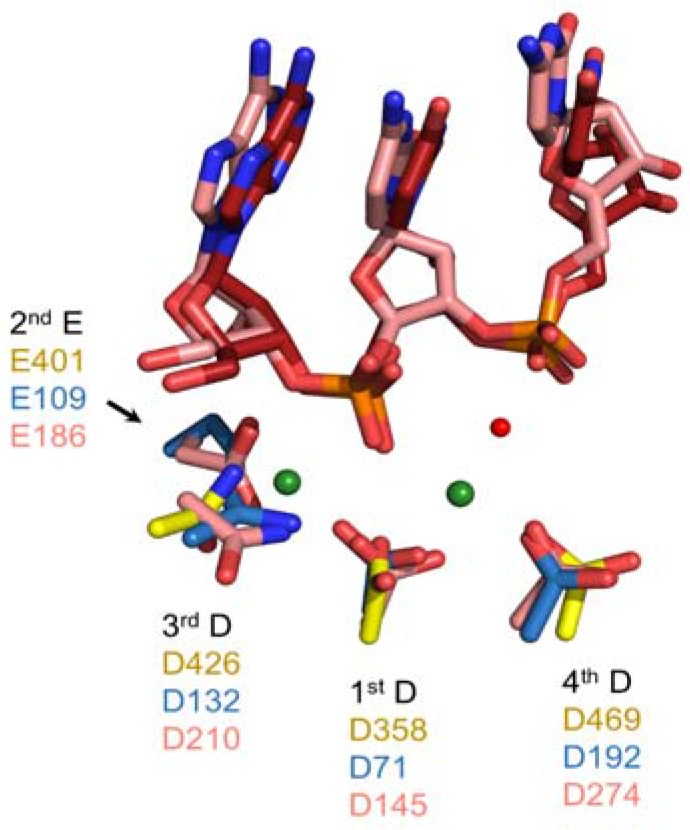
Alignment of RNase H active sites from Ty3 RT (PDB ID 4OL8, REF), *Bacillus halodurans* RNase H1 (PDB ID: 1ZB1, REF), and human RNase H1 (PDB ID: 2QK9, REF). Residue carbon atoms are shown in yellow, blue, and salmon, respectively. RNA strands from human and bacterial RNases H1 are shown in salmon and red, and two catalytic Mg^++^ ions from the Bh-RNase H1 structure are depicted as green spheres. The attacking nucleophilic water is shown as a red sphere.

Critical active site residues of the Ty3 RNase H domain are D358, E401, D426, and D469 [37,45]. These residues are superimposable with their counterparts in cellular and retroviral enzymes (Figure 6), suggesting they support a common catalytic mechanism. In biochemical assays, D358N, E401Q, and D426N substitutions eliminated RNase H activity while a D469N mutation led to its reduction [45]. The diminished effects of the D469N mutation were consistent with a prior study of the homologous residue in HIV-1 RT as well as the distinct role this acidic residue is purported to play in the 2-metal ion catalyzed model of RNase H-mediated RNA cleavage [47,48]. One distinct feature of the Ty3 RT domains is the reduced size of a loop located proximal to the active site in cellular and retroviral enzymes. As this loop harbors a conserved histidine residue (H264 in human RNase, H1 and H539 in HIV-1 RNase H) that is proposed to facilitate product dissociation following hydrolysis [47,49], its absence in the Ty3 enzyme may reduce catalytic turnover relative to the human and retroviral counterparts.

In the co-crystal structure containing an RNA/DNA hybrid, subunit A RNase H residues R441 and R445 make backbone contacts with the DNA at positions −13/−14, while subunit B residues N435 and K436 make contacts between positions −10/−11 [37]. The functional role these residues play in substrate binding and/or RNase H activity of Ty3 RT is unclear, since neither subunit is positioned for cleavage in the crystallized complex. Conversely, because homologs of Ty3 residues R473 and Y459 in HIV-1 have been shown to interact with the backbone of the RNA strand in an HIV RT-RNA/DNA co-crystal, these residues might be expected to play a similar role in a “cleavage-ready” Ty3 RT complex. R473 is well conserved among Gypsy retroelements, while mutating Y459 greatly reduces RNase H activity [45].

Homology modeling of a productive Ty3 RNase H-RNA/DNA complex indicates that a number of contacts observed to occur between cellular and retroviral RNases H and their RNA/DNA hybrid substrates would likely be missing. For example, *C*-terminal residues of β1′ in bacterial and human RNase H1 mediate contacts with 2′-OH groups on the 3′ side of active site that have been postulated as important determinants of substrate specificity [47]. Since this β-sheet is ~10 residues shorter in Ty3 RT, no such 2′-OH interactions could be established in a homologous complex. Similarly, there appears to be no Ty3 homologs of cellular and retroviral RNase H residues shown to contact the minor groove side of substrate bases (e.g., E449, N474, and Q475 of HIV-1 RNase H) [39,49]. Finally, conserved residues of the phosphate binding pocket—a motif critical for substrate recognition and DNA deformation in hybrid duplexes—have no clear homologs in the Ty3 RNase H domain [47]. Taken together, these observations suggest that, although the active site of Ty3 RNase H likely functions through a very similar mechanism to cellular enzymes, the mode of RNA-DNA binding involves fewer contacts with nucleic acid, and in particular with the DNA strand.

## 4. Structural Determinants of PPT Cleavage by Ty3 RT-Associated RNase H

**Figure 7:**
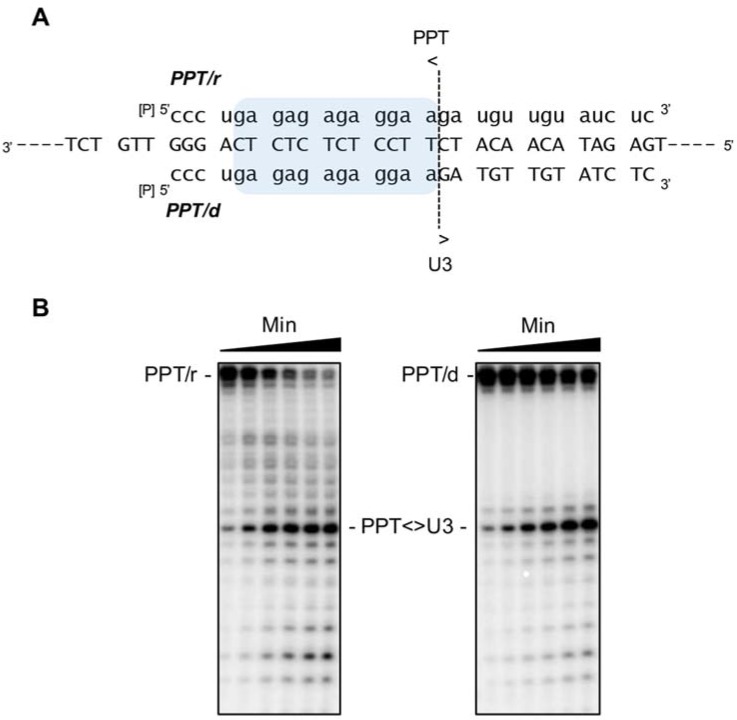
(**A**) Model RNA/DNA hybrids to illustrate the specificity of cleavage at the Ty3 PPT/U3 junction. A hybrid containing the “all-RNA” strand, PPT/r, mimics selection of the PPT 3’-OH from the RNA/DNA replication mediate during (−) strand DNA synthesis, while a hybrid containing the RNA-DNA chimera, PPT/d, mimics release of the PPT 3’-OH from nascent DNA, an obligate step following initiation of (+) strand DNA synthesis; (**B**) experimental data. For both model substrates, the position of the PPT/U3 junction has been indicated. Adapted from [50].

(+) strand DNA synthesis in LTR-retrotransposons from an RNase H-resistant PPT-containing RNA/DNA hybrid parallels mechanisms established for retroviruses. In brief, this involves (i), exposure of the PPT 3’-OH in the RNA/DNA replication intermediate; (ii), initiation of (+) strand DNA-dependent DNA synthesis; and (iii), precise removal of the RNA primer from the RNA-DNA chimera. Curiously, however, the Ty3 PPT sequence, 5′-G-A-G-A-G-A-G-A-G-G-A-A-3′ differs from its retroviral counterparts, which in general have a more homopolymeric organization (e.g., 5′-A-A-A-A-G-A-A-A-A-G-G-G-G-G-G-3′ for HIV-1). In addition, the Ty3 and HIV PPTs differ in length (12 nt and 15 nt, respectively). Despite this, model systems mimicking Ty3 PPT primer selection and its release from nascent (+) strand DNA demonstrate a high degree of precision (Figure 7), while in a heterologous system, Ty3 RT fails to recognize the HIV PPT/U3 junction [50]. Together, these observations suggest a mechanistically appropriate “fit” between the retroviral or retrotransposon polymerase and its cognate PPT drives cleavage specificity. Nucleic acid interference experiments, in combination with nuclear magnetic resonance (NMR) spectroscopy, have provided important insights into the structural basis for Ty3 PPT cleavage specificity.

**Figure 8:**
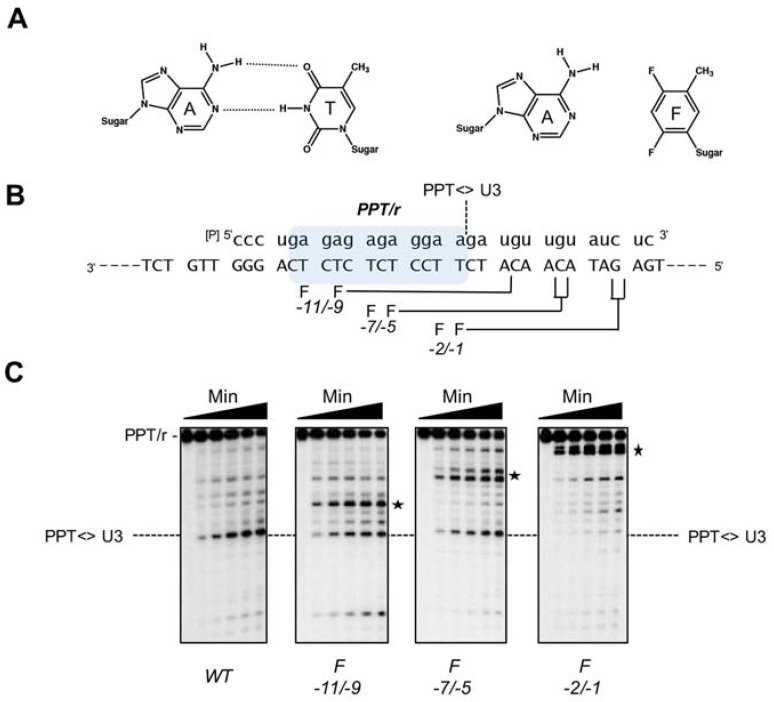
Modulation of Ty3 PPT cleavage by targeted insertion of non-polar pyrimidine isosteres. (**A**) Representation of an A:T base pair and its A:F counterpart; (**B**) Model Ty3 RNA/DNA hybrid and a summary of pyrimidine isostere mutagenesis. DNA and RNA strands are depicted in capital and small letters, respectively, and the scissile PPT/U3 junction is indicated. Base-pair numbering is relative to the PPT/U3 junction (i.e., the last base of the PPT is denoted −1). Sites of cleavage relative to the position of T-F modification in the DNA strand are indicated; (**C**) experimental data. WT, unmodified hybrid, indicating cleavage at the PPT/U3 junction. For additional panels, the position of T-F modification in the DNA strand are indicated, and the asterisk illustrates the relocated RNase H cleavage in response to these modifications. Adapted from [50,51].

The nonpolar pyrimidine mimic, 2,4-difluoro-5-methylbenzene deoxynucleoside (F, Figure 8) is isosteric with thymine, but has severely reduced hydrogen bonding capacity [51]. Its strategic insertion into the DNA strand of a Ty3 PPT RNA/DNA hybrid provided a unique means of assessing the role of hydrogen bonding without invoking major steric clashes. Most prominent among the outcomes of this strategy was the observation that a tandem −1/−2 T → F substitution quantitatively relocated cleavage specificity ~11 bp downstream (i.e., to positions +10 and +11, Figure 8). Although some specificity for the PPT/U3 junction was retained, additional dual substitutions likewise re-directed the RNase H catalytic site some 10–12 bp downstream [50]. Since the position of cleavage defined the disposition of the Ty3 RNase H domain on the hybrid, mutagenesis data indicated that local T → F-induced flexibility was “sensed” and sequestered by a structural component of Ty3 RT, leading to re-positioning of the RNase H active site. Crystallographic evidence with HIV-1 RT had suggested that several residues of its p66 thumb that were in close contact with the nucleic acid substrate could assume the role of a sensor of nucleic acid configuration [43]. Preliminary studies on Ty3 RNase H activity indicated its DNA polymerase and RNase H active sites were separated by ~13 bp of RNA/DNA hybrid [45], predicting a shorter separation distance between its thumb and RNase H domain. As indicated in Figure 3, this distance is ~10 bp, supporting such a sensor role for the subunit A thumb.

In an effort to correlate these findings with the selection of the PPT primer 3′-OH in vivo, pyrimidine isostere experiments raised the possibility that local anomalies in nucleic acid geometry, either at or upstream of the scissile junction, might also serve as recognition signals for RT positioning. A clue to this possibility was provided by NMR studies, which indicated an A- to B-transition in the +1rG sugar pucker at the Ty3 PPT/U3 junction [52]. Structurally, this local alteration in sugar pucker would alter the backbone conformation of the RNA/DNA hybrid, creating both a local distortion and, potentially, more long range kinking of the helix. An NMR structure of the junction formed at the HIV-1 (−) strand initiation site has also revealed a deoxyribose sugar switch one base step away from the junction between the tRNA primer and nascent (−) strand DNA [53]. Thus, sugar pucker switches may provide a common mechanism that contributes towards aligning RNA/DNA hybrids for correct cleavage at the RNase H active site.

Finally, as another example of subtle mechanistic differences in RTs that catalyze common steps in reverse transcription, pyrmidine isostere insertions into the DNA strand of the HIV-1 PPT have been demonstrated to similarly re-align the RNase H active site, but in this case 3–4 bp from their sites of insertion [54]. An HIV RT motif that might respond to structural anomalies is the “RNase H primer grip” (alternatively designated the phosphate binding pocket) which interacts with nucleic acid ~5 bp from the RNase H active site [38].

## 5. Conclusions and Perspectives

While the Ty3 lifecycle and RT structure share many of the features common among retroelements, numerous unique aspects of Ty3 have been highlighted in this review. The cognate minus strand primer tRNA hybridizes to distinct segments of Ty3 PBS separated by ~4800 nt in the genomic sequence, plus strand synthesis initiates multiple times from the PPT in a single reverse transcription cycle, and the PBS sequence is not perpetuated by reverse transcription of tRNA. Moreover, the RNase H domains of Ty3 RT are homologous to retroviral connection subdomains in both sequence and structural organization, and the DNA polymerase and RNase H activities of the enzyme are catalyzed by different subunits of an asymmetric homodimer. Such findings highlight not only the evolutionary commonalities and divergences among retroelements, but also the value of comparative studies in biological and biochemical research.

## References

[1] Telesnitsky A., Goff S.P., Coffin J.M., Hughes S.H., Varmus H.E. (1997). Reverse transcriptase and the generation of retroviral DNA. Retroviruses.

[2] Baltimore D. (1970). RNA-dependent DNA polymerase in virions of RNA tumour viruses. Nature.

[3] Temin H.M., Mizutani S. (1970). RNA-dependent DNA polymerase in virions of rous sarcoma virus. Nature.

[4] Le Grice S.F. (2003). “In the beginning”: Initiation of minus strand DNA synthesis in retroviruses and LTR-containing retrotransposons. Biochemistry.

[5] Nowak E., Potrzebowski W., Konarev P.V., Rausch J.W., Bona M.K., Svergun D.I., Bujnicki J.M., Le Grice S.F., Nowotny M. (2013). Structural analysis of monomeric retroviral reverse transcriptase in complex with an RNA/DNA hybrid. Nucleic Acids Res..

[6] Kirshenboim N., Hayouka Z., Friedler A., Hizi A. (2007). Expression and characterization of a novel reverse transcriptase of the LTR retrotransposon Tf1. Virology.

[7] Benzair A.B., Rhodes-Feuillette A., Emanoil-Ravicovitch R., Peries J. (1982). Reverse transcriptase from simian foamy virus serotype 1: Purification and characterization. J. Virol..

[8] Das D., Georgiadis M.M. (2004). The crystal structure of the monomeric reverse transcriptase from moloney murine leukemia virus. Structure.

[9] Perach M., Hizi A. (1999). Catalytic features of the recombinant reverse transcriptase of bovine leukemia virus expressed in bacteria. Virology.

[10] Taube R., Loya S., Avidan O., Perach M., Hizi A. (1998). Reverse transcriptase of mouse mammary tumour virus: Expression in bacteria, purification and biochemical characterization. Biochem. J..

[11] Sandmeyer S., Patterson K., Bilanchone V. (2015). Ty3, a position-specific retrotransposon in budding yeast. Microbiol. Spectr..

[12] Friant S., Heyman T., Wilhelm M.L., Wilhelm F.X. (1996). Extended interactions between the primer tRNAi(met) and genomic RNA of the yeast Ty1 retrotransposon. Nucleic Acids Res..

[13] Gabus C., Ficheux D., Rau M., Keith G., Sandmeyer S., Darlix J.L. (1998). The yeast Ty3 retrotransposon contains a 5’-3’ bipartite primer-binding site and encodes nucleocapsid protein NCp9 functionally homologous to HIV-1 NCp7. EMBO J..

[14] Ke N., Gao X., Keeney J.B., Boeke J.D., Voytas D.F. (1999). The yeast retrotransposon Ty5 uses the anticodon stem-loop of the initiator methionine tRNA as a primer for reverse transcription. RNA.

[15] Cristofari G., Gabus C., Ficheux D., Bona M., Le Grice S.F., Darlix J.L. (1999). Characterization of active reverse transcriptase and nucleoprotein complexes of the yeast retrotransposon Ty3 in vitro. J. Biol. Chem..

[16] Friant S., Heyman T., Bystrom A.S., Wilhelm M., Wilhelm F.X. (1998). Interactions between Ty1 retrotransposon RNA and the T and D regions of the tRNA(imet) primer are required for initiation of reverse transcription in vivo. Mol. Cell. Biol..

[17] Lanchy J.M., Keith G., Le Grice S.F., Ehresmann B., Ehresmann C., Marquet R. (1998). Contacts between reverse transcriptase and the primer strand govern the transition from initiation to elongation of HIV-1 reverse transcription. J. Biol. Chem..

[18] Liu S., Harada B.T., Miller J.T., Le Grice S.F., Zhuang X. (2010). Initiation complex dynamics direct the transitions between distinct phases of early HIV reverse transcription. Nat. Struct. Mol. Biol..

[19] Beerens N., Berkhout B. (2002). The tRNA primer activation signal in the human immunodeficiency virus type 1 genome is important for initiation and processive elongation of reverse transcription. J. Virol..

[20] Isel C., Westhof E., Massire C., Le Grice S.F., Ehresmann B., Ehresmann C., Marquet R. (1999). Structural basis for the specificity of the initiation of HIV-1 reverse transcription. EMBO J..

[21] Miller J.T., Ehresmann B., Hubscher U., Le Grice S.F. (2001). A novel interaction of tRNA(Lys,3) with the feline immunodeficiency virus RNA genome governs initiation of minus strand DNA synthesis. J. Biol. Chem..

[22] Aiyar A., Ge Z., Leis J. (1994). A specific orientation of RNA secondary structures is required for initiation of reverse transcription. J. Virol..

[23] Kirchner J., Sandmeyer S. (1993). Proteolytic processing of Ty3 proteins is required for transposition. J. Virol..

[24] Cristofari G., Ficheux D., Darlix J.L. (2000). The gag-like protein of the yeast Ty1 retrotransposon contains a nucleic acid chaperone domain analogous to retroviral nucleocapsid proteins. J. Biol. Chem..

[25] Karst S.M., Rutz M.L., Menees T.M. (2000). The yeast retrotransposons Ty1 and Ty3 require the RNA lariat debranching enzyme, Dbr1p, for efficient accumulation of reverse transcripts. Biochem. Biophys. Res. Commun..

[26] Lauermann V., Boeke J.D. (1994). The primer tRNA sequence is not inherited during Ty1 retrotransposition. Proc. Natl. Acad. Sci. USA.

[27] Lauermann V., Boeke J.D. (1997). Plus-strand strong-stop DNA transfer in yeast Ty retrotransposons. EMBO J..

[28] Pochart P., Agoutin B., Rousset S., Chanet R., Doroszkiewicz V., Heyman T. (1993). Biochemical and electron microscope analyses of the DNA reverse transcripts present in the virus-like particles of the yeast transposon Ty1. Identification of a second origin of Ty1DNA plus strand synthesis. Nucleic Acids Res..

[29] Nymark-McMahon M.H., Sandmeyer S.B. (1999). Mutations in nonconserved domains of Ty3 integrase affect multiple stages of the ty3 life cycle. J. Virol..

[30] Nymark-McMahon M.H., Beliakova-Bethell N.S., Darlix J.L., Le Grice S.F., Sandmeyer S.B. (2002). Ty3 integrase is required for initiation of reverse transcription. J. Virol..

[31] Wilhelm M., Wilhelm F.X. (2006). Cooperation between reverse transcriptase and integrase during reverse transcription and formation of the preintegrative complex of Ty1. Eukaryot. Cell.

[32] Le Grice S.F.J. (2012). Human immunodeficiency virus reverse transcriptase: 25 years of research, drug discovery, and promise. J. Biol. Chem..

[33] Mous J., Heimer E.P., Le Grice S.F. (1988). Processing protease and reverse transcriptase from human immunodeficiency virus type I polyprotein in *Escherichia coli*. J. Virol..

[34] Kohlstaedt L.A., Wang J., Friedman J.M., Rice P.A., Steitz T.A. (1992). Crystal Structure at 3.5 A resolution of HIV-1 reverse transcriptase complexed with an inhibitor. Science.

[35] Malik H.S., Eickbush T.H. (2001). Phylogenetic analysis of ribonuclease H domains suggests a late, chimeric origin of LTR retrotransposable elements and retroviruses. Genome Res..

[36] Rausch J.W., Grice M.K., Henrietta M., Nymark M., Miller J.T., Le Grice S.F. (2000). Interaction of p55 reverse transcriptase from the *Saccharomyces cerevisiae* retrotransposon Ty3 with conformationally distinct nucleic acid duplexes. J. Biol. Chem..

[37] Nowak E., Miller J.T., Bona M.K., Studnicka J., Szczepanowski R.H., Jurkowski J., Le Grice S.F., Nowotny M. (2014). Ty3 reverse transcriptase complexed with an RNA-DNA hybrid shows structural and functional asymmetry. Nat. Struct. Mol. Biol..

[38] Sarafianos S.G., Das K., Tantillo C., Clark A.D., Ding J., Whitcomb J.M., Boyer P.L., Hughes S.H., Arnold E. (2001). Crystal structure of HIV-1 reverse transcriptase in complex with a polypurine tract RNA:DNA. EMBO J..

[39] Lapkouski M., Tian L., Miller J.T., Le Grice S.F., Yang W. (2013). Complexes of HIV-1 RT, NNRTI and RNA/DNA hybrid reveal a structure compatible with RNA degradation. Nat. Struct. Mol. Biol..

[40] Lapkouski M., Tian L., Miller J.T., Le Grice S.F., Yang W. (2013). Reply to “Structural requirements for RNA degradation by HIV-1 reverse transcriptase”. Nat. Struct. Mol. Biol..

[41] Bibillo A., Lener D., Klarmann G.J., Le Grice S.F. (2005). Functional roles of carboxylate residues comprising the DNA polymerase active site triad of Ty3 reverse transcriptase. Nucleic Acids Res..

[42] Sawaya M.R., Pelletier H., Kumar A., Wilson S.H., Kraut J. (1994). Crystal structure of rat DNA polymerase beta: Evidence for a common polymerase mechanism. Science.

[43] Bibillo A., Lener D., Tewari A., Le Grice S.F. (2005). Interaction of the Ty3 reverse transcriptase thumb subdomain with template-primer. J. Biol. Chem..

[44] Koshkin A.A., Singh S.K., Nielsen P., Rajwanshi V.K., Kumar R., Meldgaard M., Olsen C.E., Wengel J. (1998). LNA (locked nucleic acids): Synthesis of the adenine, cytosine, guanine, 5-methylcytosine, thymine and uracil bicyclonucleoside monomers, oligomerisation, and unprecedented nucleic acid recognition. Tetrahedron.

[45] Lener D., Budihas S.R., Le Grice S.F. (2002). Mutating conserved residues in the ribonuclease H domain of Ty3 reverse transcriptase affects specialized cleavage events. J. Biol. Chem..

[46] Nowotny M. (2009). Retroviral integrase superfamily: The structural perspective. EMBO Rep..

[47] Nowotny M., Gaidamakov S.A., Crouch R.J., Yang W. (2005). Crystal structures of RNase H bound to an RNA/DNA hybrid: Substrate specificity and metal-dependent catalysis. Cell.

[48] Rausch J.W., Le Grice S.F. (1997). Substituting a conserved residue of the ribonuclease H domain alters substrate hydrolysis by retroviral reverse transcriptase. J. Biol. Chem..

[49] Nowotny M., Gaidamakov S.A., Ghirlando R., Cerritelli S.M., Crouch R.J., Yang W. (2007). Structure of human RNase H1 complexed with an RNA/DNA hybrid: Insight into HIV reverse transcription. Mol. Cell.

[50] Lener D., Kvaratskhelia M., Le Grice S.F. (2003). Nonpolar thymine isosteres in the Ty3 polypurine tract DNA template modulate processing and provide a model for its recognition by Ty3 reverse transcriptase. J. Biol. Chem..

[51] Guckian K.M., Krugh T.R., Kool E.T. (2000). Solution structure of a nonpolar, non-hydrogen-bonded base pair surrogate in DNA. J. Am. Chem. Soc..

[52] Yi-Brunozzi H.Y., Brabazon D.M., Lener D., Le Grice S.F., Marino J.P. (2005). A ribose sugar conformational switch in the LTR-retrotransposon Ty3 polypurine tract-containing RNA/DNA hybrid. J. Am. Chem. Soc..

[53] Szyperski T., Gotte M., Billeter M., Perola E., Cellai L., Heumann H., Wuthrich K. (1999). NMR structure of the chimeric hybrid duplex r(gcaguggc).R(gcca)d(CTGC) comprising the tRNA-DNA junction formed during initiation of HIV-1 reverse transcription. J. Biomol. NMR.

[54] Rausch J.W., Qu J., Yi-Brunozzi H.Y., Kool E.T., Le Grice S.F. (2003). Hydrolysis of RNA/DNA hybrids containing nonpolar pyrimidine isosteres defines regions essential for HIV type 1 polypurine tract selection. Proc. Natl. Acad. Sci. USA.

